# Human Heads and Their Covering

**Published:** 1853-11

**Authors:** 


In introducing to our readers the following remarks of the editor of the Boston Medical and Surgical Journal, we have a due sense of the importance of the subject. Since the publication, in this journal, of the article on "Hats and Baldness," by the senior editor, we have looked with a decided antipathy on those abominable inventions, yclept hats. They are an ungraceful combination of the properties of a starch bandage and a sap-bucket—a segment of a stove-pipe is not more unyielding, or less ornamental. A good head of hair is the glorious garniture of a perfect shape—the crowning capital of a splendid column. Without it man is a round topped hitching post. This is a serious matter. It is no joke to lose one's hair; and he who ridicules the bald is devoid of human sympathies—he would sneer at a cripple. But not only our hair must perish from our craniums like leaves in autumn to please immitigable fashion, but our wits must be cramped, and our intellects made to grow to fill a certain space prescribed by some Genin, or Leary, like a cucumber placed in its infancy in a phial, "cabined, cribbed, confined." S. B. H.

*Human Heads and their Covering.*—In the lifetime of Spurzheim, and subsequently, when Mr. Combe, the learned Scotch phrenologist, was in this country, the leading conversation of the day related to the shape and magnitude of heads. A man without a head of the recognized capacity, soon lost the acquaintance of caput critics. Nothing short of a large skull, however empty, had any prospect of success. A great head is unquestionably the receptacle of more apparatus than a small one. Still, admitting the proverb to be true, that a "big head has little wit, and a little head not a bit," it becomes a puzzling consideration at times to determine which of the two is best for an ordinary individual. Our national fashion of wearing stiff, hard hats, is unfavorable to a noble cerebral development. While cloth, canvas and fur caps yield to the pressure of outward growth, hats, on the contrary, act as hoops, interfering with that degree of lateral expansion necessary in the economy of nature, who, in her steady purpose to furnish a man, in common parlance, fit for market, is thus interrupted. In Germany, caps are predominant; and where the hat is substituted with them, it is usually soft and pliable as a turban. Germans have enormous heads, with corresponding powers. They excel in all departments of knowledge; and in music, poetry, and the fine arts, who are their superiors? School-boys require a head-covering that has reference to the daily enlargement of the cranium. At least, we should give it a chance to grow, and on no consideration interfere with its expansion. Parents should look to this matter. It would be lamentable to lose, for the want of a woollen cap, any talent existing in embryo, that with it may have ripened into a philosopher, a general, or something else equally elevated! Proceeding upon the current doctrine of the phrenologists, we may cultivate human heads—diminishing or enlarging them, according to the varying fashions of the day. But to be serious, our heavy hats ought not to be worn by any one. They are the most awkward and uncomfortable coverings the arbitrary laws of society ever countenanced. Turks and Arabs are sensible people, in respect to covering the head. A turban is superior to every other device. In health or sickness, it is a luxury. Had those Mahommedan bigots, with their well-developed heads, been placed under proper mental discipline, and taught like Christians, they would have been surprisingly intellectual, and have demonstrated, among the best specimens of Asiatic blood, that a great head indicates great mental capacity.—*Boston Medical and Surgical Journal.*

